# Understanding frailty and its opposites from community-dwelling older peoples’ perspectives: A phenomenological qualitative study

**DOI:** 10.1016/j.ijnsa.2024.100238

**Published:** 2024-09-06

**Authors:** Rianne DJ Golbach, Nanda Kleinenberg-Talsma, Fons van der Lucht, Johannes SM Hobbelen, Harriët Jager-Wittenaar, Evelyn J Finnema

**Affiliations:** a Department of Science in Healthy Ageing and Healthcare (SHARE), University of Groningen, University Medical Center Groningen, Groningen, the Netherlands; b Research Group Healthy Ageing, Allied Health Care and Nursing, Hanze University of Applied Sciences, Groningen, the Netherlands; c FAITH research, Groningen/Leeuwarden, the Netherlands; d National Institute of Public Health and the Environment, Centre for Health and Society, Bilthoven, the Netherlands; e Aletta Jacobs School of Public Health, Groningen, the Netherlands; f Department of General Practice and Elderly Care Medicine, University of Groningen, University Medical Center Groningen, Groningen, the Netherlands; g Department of Gastroenterology and Hepatology, Dietetics, Radboud University Medical Center, Nijmegen, the Netherlands; h Vrije Universiteit Brussel, Faculty of Physical Education and Physiotherapy, Department Physiotherapy and Human Anatomy, Research Unit Experimental Anatomy, Belgium; i Department of Health Sciences, Section of Nursing Research, University of Groningen, University Medical Center Groningen, Groningen, the Netherlands; j Research Group Nursing Diagnostics, Hanze University of Applied Sciences, Groningen, the Netherlands; k Research Group Living, Wellbeing and Care for Older People, NHL Stenden University of Applied Sciences, Leeuwarden, the Netherlands

**Keywords:** Ageing, Autonomy, Cognition, Frailty, Perspectives, Resilience, Vitality

## Abstract

**Background:**

the global population is ageing. As older people become more susceptible to frailty, an increase in frailty prevalence is also expected. Although frailty has been defined before in research, older peoples’ perceptions of frailty do not always coincide with those used in research or medical settings. Further exploring community-dwelling older people's viewpoints regarding frailty is essential for tailored care and policy.

**Aim:**

the aim of this study was to explore the perspectives of Dutch community-dwelling older people regarding frailty and its opposing concepts.

**Methods:**

a phenomenological qualitative study was conducted for which we carried out semi-structured interviews with independently living older people aged ≥65. Following the interviews, the participants filled out the Tilburg Frailty Indicator.

**Results:**

the different domains of frailty: ‘physical’, ‘psychological’, and ‘social’, were recognized by participants. In addition, other aspects, such as financial capacity and digital functioning, have been identified. Four aspects of the meaning of frailty were identified in the category of other frailty definitions: ‘dependency’, ‘frailty as getting hurt’, ‘frailty as prone to deterioration’, and ‘frailty as experiences of loss and sacrifice’. Participants also described the opposites of frailty, which could also be distinguished according to the ‘physical’, ‘psychological’, and ‘social’ domains. In addition, participants mentioned the following concepts as opposing frailty: ‘vitality’, ‘resilience’, ‘independence’, ‘autonomy’, and ‘ambition’.

**Conclusion:**

we found that frailty and its opposites share similar aspects, including physical, psychological, and social dimensions. Additionally, older people perceived cognition as an essential aspect of frailty. The psychological dimension seemed more dominant in concepts opposed to frailty, which raises opportunities to focus on the positive aspects and build on older people's (psychological) capabilities in managing frailty and its consequences. Based on these findings, policymakers and care professionals should consider the perspectives of older people regarding frailty and its opposing concepts.

What is already known about the topic

• Frailty perspectives of older people do not correspond with frailty definitions.

• A more positive, constructive approach to the management and treatment of frailty is necessary.

**What this paper adds**

• The concept of frailty encompasses digital and financial aspects next to frailty dimensions, such as physical, psychological, and social dimensions.

• Community-dwelling older people described cognitive frailty as an essential part of frailty.

• Psychological aspects were dominant in describing the opposites of frailty; e.g., vitality, resilience, and autonomy.

Alt-text: Unlabelled box

## 1 Introduction

The global population is ageing; while in 2021, one in 10 people were aged ≥65, in 2050 this is expected to be one in six people (United Nations Department of Economic and Social Affairs, 2023). Several demographic trends underlie the rise in the ageing population: fertility rates decline, leading to a smaller number of younger people, while life expectancy increases, leading to older people living longer (United Nations Department of Economic and Social Affairs, 2023). As older people become more susceptible to frailty, an increase in frailty prevalence is expected as well (Hoogendijk et al., 2019; Rockwood and Howlett, 2018). As a result, the need for health and social care also tends to increase (Baâdoudi et al., 2023). Frailty is a syndrome that challenges older individuals, the healthcare system, and public health (Doody et al., 2023; Hoogendijk et al., 2019).

Frailty has been defined in several ways, from unidimensional, focusing on someone's physical state, to multidimensional, incorporating other dimensions (De Donder et al., 2019). A common approach to frailty was developed by Fried, focusing on five markers of an individual's physical state; i.e., unintentional weight loss, reduced handgrip strength, decreased endurance, reduced walking speed, and reduced level of activity (Fried et al., 2001; Sobhani et al., 2021). Moving away from this unidimensional approach, several multidimensional approaches have been utilized in recent years. One of these multidimensional approaches is the accumulation of deficits (Rockwood and Mitnitski, 2007). The more deficits (e.g., symptoms, signs, or diseases) are present in an individual, the higher the likelihood of being frail; i.e., the higher the risk of adverse outcomes (Rockwood and Mitnitski, 2007). In the conceptual frailty framework of Gobbens, frailty is seen as a dynamic state in which a person experiences loss in one or several health domains; e.g., physical, social, or psychological (Gobbens et al., 2010b).

However, older people's perceptions of the concept of frailty do not always coincide with the definitions used in research or medical settings (Archibald et al., 2020; Schoenborn et al., 2018). Several qualitative researchers have revealed that older people see frailty as an inevitable part of ageing, which is irreversible (Escourrou et al., 2019; Schoenborn et al., 2018). Archibald et al. (2020) revealed in a qualitative study among older people, that frailty is often perceived as vague, negative, and associated with end-of-life (Archibald et al., 2020). It was also found that the term frailty carried negative associations when used by health professionals (Escourrou et al., 2019).

These approaches to frailty, whether unidimensional or multidimensional, focus on the negative side of health: deficits, loss, and decline. In current research, however, there is a trend toward a more positive approach to frailty communication and management. For example, in a recent Delphi study, older people in the Netherlands indicated that they did not use or like negative terms, such as frail, fragile, or brittle, but instead preferred positive terms concerning older age, such as independence, resilience, or vital (Deleted for blinded review). Also, researchers pointed towards balancing factors for frailty, emphasizing the deficits and the assets available to an individual (De Donder et al., 2019). This is consistent with the advice that a more positive framing of frailty could fortify strategies for identifying and managing frailty (Archibald et al., 2020).

The perceptions and experiences of community-dwelling older people regarding frailty are essential for health professionals and policymakers to develop tailored care or policy plans. Furthermore, gaining insight into what opposes the frailty concept is equally important in light of a more positive approach to frailty communication and management. Therefore, with this study, we aimed to explore the perspectives of Dutch community-dwelling older people regarding frailty and its opposing concepts.

## 2 Methods

### 2.1 Study design

A phenomenological qualitative study was conducted for which we carried out semi-structured interviews with older people. Interviews were followed by a frailty questionnaire, the Tilburg Frailty Indicator (Gobbens et al., 2010b). This study was part of a larger qualitative study on the perspectives of frail and non-frail older people on frailty (also described in (Deleted for blinded review) and (Deleted for blinded review)).

### 2.2 Sample

A convenience sample of older people aged ≥65 and living independently was recruited. Recruitment was purposeful by contacting older people who previously participated in a study on the terminology older people use concerning frailty (Deleted for blinded review) and by contacting the partners from the research- and practice network the researchers collaborate with (e.g., Denktank 60+ Noord). In addition, the researchers actively approached older people in their network (e.g., relatives or by contacting relatives working in senior care). Initial recruitment resulted mainly in higher-educated older people in good health. Therefore, additional purposeful recruitment took place to select more diverse participants. The researchers contacted professionals in home care organizations, physiotherapist practices, and a local social welfare organization that works with frail and non-frail older people. When data saturation was reached, indicated by the lack of new information, recruitment was ended.

### 2.3 Data collection

Interviews were conducted at participants' homes or, when preferred by participants, in a (semi) public location. Face-to-face interviews were the preferred data-collection method, although due to COVID-19 restrictions or preferences of participants, interviews could also be held online (Microsoft Teams meeting). Interviews were conducted by one of the two primary researchers (Author 1 and Author 2).

We worked with a pre-determined topic list drafted by the primary researchers and checked by the research team. The topic list was designed based on the research questions and the conceptual model of frailty by Gobbens and colleagues (Gobbens et al., 2010b). It followed a funnel model; the interviews started with general questions about ageing and daily activities and were followed by more specific questions about frailty, its conceptualisation, and concepts opposing frailty. Before conducting the interviews, two pilot interviews were performed to check the topic list and the flow of the interviews. Minor adjustments were made based on these pilot interviews and during data collection; for example, in the sequence of questions or specific follow-up questions. The interviews were conducted in Dutch, quotations were translated to English by a bilingual person. During the pilot interviews, we noted participants' difficulty in dealing with the concept of frailty. In Dutch, we use one word (i.e.,‘kwetsbaarheid’) for both frailty and vulnerability; ‘kwetsbaarheid’ has different meanings and is often used in different contexts. This could have resulted in different conceptualisations of ‘kwetsbaarheid’ and hence ‘frailty’. The interviews were conducted between November 2021 and August 2022 and lasted between 30 and 120 min. Following the interviews, the participants filled out the Tilburg Frailty Indicator (Gobbens et al., 2010a), a concise questionnaire to determine their frailty status and to collect socio-demographic information.

### 2.4 Reflexivity

The two primary researchers were both experienced in ageing research. Author 2 has a background in sociology and Author 1 in human movement sciences. Both researchers received interview training before the data collection process. To minimize the impact of the researchers during data collection and to avoid prejudices, participants known by one researcher were interviewed by the other, except for two participants who were close relatives of one researcher. They felt more comfortable being interviewed by their relative. In addition, during or after the interviews, the interviewers took notes to express their observations, thoughts, or considerations.

### 2.5 Data analysis

The interviews were audio recorded and transcribed verbatim. To gain familiarity with the data, transcriptions were partly conducted by the primary researchers and partly by a transcription company. The data were coded and analysed using ATLAS.ti 23. The data analysis was based on the steps of thematic analysis (Braun and Clarke, 2022). First, data familiarisation took place by transcribing and reading the transcripts. Second, a combination of deductive and inductive code development was used to create the first version of the codebook (Hennink et al., 2020) Codes were taken from literature and the conceptual frailty model by Gobbens (Gobbens et al., 2010b). The two primary researchers (Author 1 and Author 2) independently read and coded the same two transcripts and inductively developed additional codes. These results were discussed thoroughly and taken as the basis for the codebook. After that, the primary researchers again coded a third transcript and discussed the results. If needed, adjustments were made to the codes, and the definitive codebook was established. The coding was continued separately. In case of doubts or lack of clarity, issues were discussed between the two researchers. Third, initial themes were generated based on the thick descriptions developed by the researchers and discussed with the research team. In addition, one participant was consulted to discuss the categorisation of the concept of frailty. After that, in discussion with the research team, the themes were further explored and refined. After this step, final considerations of the themes and their context were discussed between the two primary researchers before writing the findings.

The frailty scores and socio-demographic information obtained by the Tilburg Frailty Indicator (Gobbens et al., 2010a) were processed and analysed with Qualtrics XM and IBM SPSS statistics (version 28.0.1.1).

### 2.6 Ethical considerations

The study was approved by the Ethical Advisory Committee of the Hanze University of Applied Sciences (heac.2021.020). Before the interviews, written informed consent was obtained from all participants.

### 2.7 Trustworthiness

Additionally, some actions were taken to enhance the study's trustworthiness. Member checks were conducted, in which summaries of the transcripts were sent to participants. No additional remarks or comments were given based on these summaries. In addition, researcher triangulation took place through weekly meetings with the two primary researchers and regular meetings with the research team.

## 3 Findings

The interviews were conducted with 36 participants, of whom the majority were female. The oldest participant was 93 years old. Fifteen participants were considered frail based on the outcomes of the Tilburg Frailty Indicator (score ≥5), and 21 participants were considered non-frail (score <5). In Table 1, characteristics of the participants are presented.

**Table 1:** Participant characteristics (*N* = 36).

		**N**
**Sex (female)**		24 (66,7 %)
**Age**		77.8 ± 7.1 (65–93)
**Marital status**		
Married		23
Unmarried		2
Divorced		4
Widowed		7
**Birth Country**		
The Netherlands		34
Chile		1
Sweden		1
**Educational level**		
None or elementary school		3
Secondary professional education		14
High school or university		19
**Monthly income (euros)**		
≤600		1
601 – 900		1
901 – 1200		1
1201 – 1500		2
1501 – 1800		8
1801 – 2100		3
≥2101		20
**Healthy lifestyle**		
Healthy		31
Not healthy, not unhealthy		5
**≥ Two diseases or chronic conditions**		18
**Frail^a^**		15

Notes: *^a^*≥ 5 score on the Tilburg Frailty Indicator (TFI).

Twenty-eight interviews were face-to-face, and five were held online (Microsoft Teams) due to participants’ preferences or COVID restrictions. Nearly all interviews were conducted individually; three were conducted with couples (husband/wife).

Themes were subdivided into two categories: *1. Conceptualisation of frailty,* and *2. Concepts opposing frailty*. Themes identified under *1. Conceptualisation of frailty* were *frailty domains; e.g., physical, psychological (and cognitive), social, and other aspects of frailty (with financial capacity and digital functioning)* and *other frailty definitions;* e.g., *dependency, getting emotionally hurt, prone to deterioration,* and *experiences of loss and sacrifice*. In addition, themes identified under the *2. Concepts opposing frailty* were *physical, psychological, social,* and *other aspects opposing frailty*; e.g., *vitality, resilience, independence, autonomy, ambition,* and *self-control.*

## 4 Frailty domains

The different domains of frailty - physical, psychological, and social - as described in the literature (Gobbens et al., 2010b) and distinguished in measurement instruments, policy, and research were recognized by participants. In addition, other themes were identified and placed under external aspects, such as financial capacity and digital functioning.

### 4.1 Physical

Physical functioning was considered a frailty domain, as often mentioned by participants. They related physical distress to health statuses, such as illnesses or overall health functioning. In addition, specific health problems were also mentioned as factors related to the physical domain of frailty. Participants mentioned fatigue, energy loss, vision or hearing decline, osteoarthritis, or mobility restrictions due to walking, running, or cycling difficulties. Another frequently mentioned aspect was a higher risk of falls and the physical consequences of experiencing a fall, such as breaking something. As respondent described:

“When you fall, you are more likely to break something: a leg, an arm, or whatever. You are more likely to break bones than when you were young. (…) You become clumsier with walking, I think. Look, when you are young, you jump over things more easily than when you become old.” (male, 74, non-frail)

### 4.2 Psychological

Participants mentioned psychological aspects of frailty next to the physical domain. The psychological aspects were related to specific emotions associated with experiencing deterioration, such as anxiety, insecurity, distrust, worrying, or being easily touched. In addition, older people mentioned they were struggling to accept that they could no longer do everything they used to, that their role in life was changing, and that they could no longer participate in society fully. These experiences were related to becoming increasingly careful or even anxious. One of the participants shared:

“In the past, I used to be fearless. Now, I am more cautious. And that is the only way I have become vulnerable. I wouldn't go out alone in the dark anymore.” (female, 74, frail)

Additionally, the realization that life is in its latest stage or that friends or relatives are in their final phase was mentioned.

Another aspect of psychological frailty emphasized by participants was a decline in cognitive functioning, indicated by memory loss, difficulty in keeping up with social developments, and features of dementia. Other examples were forgetting things, losing control over oneself, not getting one's thoughts in order, or the inability to manage financial issues digitally. One of the respondents was aware that she was in the early stages of dementia. As she put it:

“It sounds strange, but my ‘me’ is gone. I am dependent on the people who still are… different than me. I am going downhill a bit, and you are still in this world. And I cannot fit in there.”(female, 80, frail)

Others referred to people around them who had experienced dementia or who were deteriorating. Dementia appeared to be a disastrous outcome for participants and was something they wanted to avoid at all costs. Feelings of extreme anxiety or frightfulness often accompanied it.

### 4.3 Social

Besides to physical and psychological aspects, social aspects were described as a domain in which frailty manifests itself. In addition, social aspects were not only described as characteristics of frailty but also as risk factors. The importance of having a network on which one can rely was mentioned; a small network, no family, no children, no neighbours, no church, or having none of the networks nearby can make someone frail. When people age, it seems more challenging to maintain a network. A diminished network can result from losing relatives, friends, or partners or when an established group breaks or changes. Being alone or ending up alone was experienced as a risk factor for frailty.

“Well, I think if you end up alone… that you are more frail than when you live together as husband and wife.” (male, 74, non-frail)

On the other hand, reliance on and support of one's social network was also mentioned as something to build on, accompanied by feelings of joy. Children were mentioned in this context. Some relied heavily on their children, whereas others emphasized the children had their own lives and one had to see whether they were available.

“Another part of frailty is that your children have their own lives, that you are no longer the most important person in their lives. (…) You have to find a way to keep in touch and be there for them and hope they want to be there for you.” (female, 74, frail)

Another social aspect described was being unable to keep up, for example, in social activities or conversations. This can be due to physical deterioration, such as mobility limitations or hearing loss. Participants differed in how much they were bothered by this.

*Loneliness* was described in the context of maintaining a social network, getting support, and the loss of close ones, such as friends or partners. But loneliness was also described as lacking a social network and having no one to lean on.

“Perhaps it is different for everyone, but for me, it is if you… if you don't have a network, I guess. No family, no church, no neighbours. That you don't have anyone. (…) I think it depends on your social status how frail you are.” (female, 75, non-frail)

However, it also contained an emotional aspect, feeling lonely, as described by this woman:

“And that's where the social circle, the circle you can share memories with, also gets smaller and smaller. And I do find that very difficult. So because of that, it also becomes a form of loneliness, because you can't share anymore. That's a closed chapter. That is what I find difficult about being old at the moment.” (female, 73, non-frail)

### 4.4 Other aspects of frailty

Participants mentioned financial capacity and digital functioning as important aspects of frailty.

Limited financial capabilities, feeling like one had fewer financial resources than one needed, not having a healthy financial situation, or falling short of money were described as limiting physical, social, or psychological functioning. Examples were a lack of possibilities to participate in physical or social activities or feelings of worrying and discomfort it might evoke.

Besides financial problems, limited digital skills were also described as an aspect of frailty. Participants mentioned they experienced difficulties logging in to specific web pages or managing their finances online. In addition, digital functioning was described as being unable to keep up with digital developments, which can also evoke feelings of discomfort. This seemed related to the notion of having to rely on others and ask for help. In addition, difficulties in understanding and working with computers in general made participants feel frail in a certain way. It could interfere with keeping up with society and maintaining social contacts.

“If you don't [keep up with digital developments], you can get isolated from society.” (male, 75, non-frail)

## 5 Other frailty definitions

Participants described that they found frailty challenging to conceptualize or define. Their definitions were based on their experiences or experiences with people around them. Four aspects of the meaning of frailty could not be placed under a single domain and were identified in the category of *other frailty definitions: dependency,* frailty as *getting hurt*, frailty as *prone to deterioration*, and frailty as *experiences of loss and sacrifice*.

### 5.1 Dependence

Dependence was mentioned by participants as an indicating factor of frailty. Dependence was most often described concerning others, such as being dependent on others, relying on the help of others, or needing to ask for help; for example, in self-care, needing help with washing, dressing up, or cleaning the house. For most people, the feeling of being dependent on others is unpleasant and therefore evokes resistance. Dependency detracts from a sense of self-worth and control over one's life. Being dependent on others increases the level of frailty.

“They [acquaintances] are also not allowed to drive a car at the moment, so then you really become dependent on formal care, and on help, from neighbours, caregivers, you name it. And I think that's what makes you frail.”(female, 72, non-frail).

In addition, dependency was also characterized by being unable to do what one wanted, not being able to take care of oneself, and not being independent, also in one's mind. Dependence was often indicated by physical limitations; e.g., mobility restrictions. One's world gets smaller, literally and figuratively.

### 5.2 Getting emotionally hurt

Some participants described frailty as getting emotionally hurt, which revealed itself in relation to others. They experienced this positively or negatively. It showed a particular sensitivity that could be pleasurable, as this woman described:

“I can be very moved by what someone says. But that is a vulnerability that is also pleasant. A vulnerability that touches you.” (female, 72, frail)

On the other hand, it could be unpleasant, for example, when someone felt aggrieved by what others said or did.

### 5.3 Prone to deterioration

Another aspect recalled by participants was susceptibility to deterioration. The susceptibility to deterioration manifested itself along several facets of life, such as susceptibility to physical decline (for example, to falls, illness, and fractures) but also prone to losses in their social network or the idea that only something small has to happen to break down. Knowing that frailty entails a certain degree of susceptibility to all kinds of deterioration mainly stirred feelings of anxiety. Anxiety about the consequences if something happened was worse than when one was younger or less frail. Also, this susceptibility was perceived as confronting, being unable to do what one could before or having to think more carefully about the consequences of one's actions.

“You realise you are at an age where it could be over tomorrow. When you're fifty, you don't think about that. But we have lost many friends. They die, they just die. They get dementia. They hear one day they have cancer and a month later they are dead.” (female, 75, non-frail)

Susceptibility to deterioration revealed a precarious balance in which minor events or experiences might result in adverse outcomes in one or more frailty domains. Some participants saw this as an accumulation of deficits. An accumulation of deficits indicates a tipping point; when multiple deficits are present in one or multiple domains, the likelihood of becoming frail increases. As this woman described:

“You get an accumulation of discomforts, physical discomforts, but it can also be mental, that you deteriorate mentally [when you age]. And for me, the tipping point is when I need help every day - either domestic help, for example with getting dressed, physically, or indeed mental help.” (female, 72, non-frail)

### 5.4 Experiences of loss or sacrifice

Another way frailty was described is by experiencing loss in various domains of human functioning or feelings of sacrifice in daily life.

“For me, frailty is losing more and more. Of yourself, of your environment, of your activities.” (female, 71, frail)

In social functioning, experiences of loss were described as losing loved ones, becoming alone, or being unable to keep up with others. In addition, in psychological functioning older people described loss as losing oneself, not knowing oneself anymore, or feeling lost. These feelings could also manifest when experiencing loss in physical functioning; for example, due to mobility restrictions and physical deterioration resulting in functional limitations, such as no longer being able to drive a car. These limitations or combinations of sacrifice on multiple facets of life made an individual's surroundings feel smaller.

## 6 Concepts opposing frailty

In the interviews, participants were also asked what they perceived as the opposite of frailty and how they would describe this. Similar to the frailty aspects, aspects related to the opposite of frailty could also be distinguished according to different domains, such as physical, psychological, and social.

### 6.1 Physical

Feeling strong, being healthy, feeling fit, and being active were physical descriptions opposing frailty. Participants explained this as being active and doing what one likes and values, such as visiting others or just driving somewhere.

### 6.2 Psychological

Participants emphasized the psychological aspects of concepts opposing frailty. A positive attitude, not victimizing themselves, and adjusting to what comes one's way was essential. One of the participants described a non-frail person as follows:

“Someone who takes good care of himself, who doesn't see him or herself as a victim. That attitude of ‘why does it always have to happen to me?’. Then it's over.”(female, 93, frail)

In addition, adapting to one's age or situation was also part of the psychological aspects.

### 6.3 Social

Regarding social aspects, participants explained the importance of being close to others, trusting others, and having a social network.

“Being close, closer again to other people. Things are so cold and distant. (…) Being fully present in life, that is it for me.” (female, 71, frail)

### 6.4 Other aspects opposing frailty

Apart from the aspects related to the domains above, participants mentioned the following concepts as opposing frailty: vitality, resilience, independence, autonomy, and ambition.

**Being vital** was an often-mentioned term to oppose frailty. Participants explained this as being in shape, both physically and mentally. A vital person was described as someone active and involved in life. A female participant recognized a vital older adult by his or her appearance and behavior:

“How they behave. Usually also physically. Or when someone is at an advanced age and still gives an outstanding lecture. Something like that.” (female, 73, non-frail)

In addition, participants described **resilience** as the opposite of frailty, explicated as standing firm, being able to cope, and adjusting after setbacks in life.

Furthermore, **independence** was described as the opposite of frailty. Being independent and self-reliant, not having to depend on others, was seen as key in this.

“It has to do with independence, wanting or being able to do it yourself, as much as possible.” (female, 70, non-frail)

Another aspect mentioned by participants as the opposite of frailty was **autonomy**. Having the autonomy to do the things that one prefers or values and having self-control in life was deemed essential. A female, non-frail participant explained:

“That I can live my life in a way that suits me.” (female, 76, frail)

Next, **ambition** was described as opposing frailty, not in a professional sense but because of the need to have meaningful activities and contribute to society.

## 7 Discussion

In this paper frailty and its opposites were described from the perspectives of Dutch, community-dwelling older people. The concepts and viewpoints regarding frailty and its opposing concepts, as described by older people, raise opportunities to manage frailty and its consequences constructively. In particular, older people emphasized their psychological capacity represented in positive concepts, such as autonomy, vitality, and resilience. Frailty was perceived as a multidimensional concept, including physical, psychological, and social aspects. In addition, financial security and digital abilities were considered essential aspects affecting frailty. Furthermore, frailty definitions were described as ‘dependence’, ‘getting emotionally hurt’, ‘prone to deterioration’, and ‘experiences of loss and sacrifice’. Moreover, the opposites of frailty, according to older people, reflect similarities to frailty, as shown in the classification in physical, psychological, and social dimensions, with major attention to psychological concepts, such as vitality, resilience, and independence.

Older people emphasized a broad view of frailty and its related aspects, in which risk factors, indicators, and consequences of frailty mutually affected one another. Generally, older people's descriptions of frailty as a dynamic and multi-dimensional construct were in line with recent studies on frailty definitions (Sobhani et al., 2021). However, their broader view on frailty raises challenges in practice to discuss what frailty entails on personal levels since understandings might differ from the perspectives of social or care professionals. Previously, researchers showed this complexity and advocated an approach in which multiple perspectives can be combined. Attention should be given to the context and characteristics of older individuals (Deleted for blinded review; Sobhani et al., 2021).

As we have exposed in the current study, the focus on cognitive aspects is notable. In contrast to our findings, other researchers have found that cognitive aspects were mainly categorized in the psychological domain (Gobbens et al., 2010b; Sobhani et al., 2021). However, during the interviews, cognitive decline received significant attention from older adults, implying that it is an essential aspect of frailty for them. Older adults described cognitive aspects as characteristics of frailty, in which the following features were prevalent: experiences of memory loss, difficulties in keeping up with societal developments, and features of dementia. We argue that cognitive frailty might be classified as a separate domain or an aspect that can be distinguished from other domains since it reveals characteristics of someone's cognitive status and decline. This aligns with previous studies; in the Comprehensive Frailty Assessment Instrument, the cognitive domain was added to the measurement to enhance its comprehensiveness (De Roeck et al., 2018). In addition, in the report on older people and frailty from the National Institute for Public Health and the Environment, cognitive aspects were classified as separate next to the physical, psychological, and social domains in the integral conceptual model of Gobbens (Rijksinstituut voor Volksgezondheid en Milieu, 2015). Designating cognitive aspects as a separate category increases understanding of what frailties manifest in older people and how they mutually interact. With repositioning cognitive aspects of frailty, opportunities arise to adequately manage and treat frailty in older people; for example, by focusing on interventions to restore cognitively healthy lifestyle habits (Facal et al., 2019).

Next to the frailty domains (e.g., physical, psychological, social, and cognitive frailty), older adults mentioned financial capacity and digital functioning as aspects of frailty. These were also found to be important factors of frailty in previous studies, in which researchers showed that concerns about financial capacity or experiencing financial limitations might affect the risk of frailty or its course (Peek et al., 2012). On the one hand, financial concerns can worsen frailty characteristics in other domains; for example, the inability to participate in social or physical activities. On the other hand, it can be a balancing factor, as financial resources offer possibilities to purchase goods and services (Dury et al., 2018). In addition, digital functioning concerned older adults; they described having difficulty keeping up with digital developments, which was also found to harm their frailty balance (Dury et al., 2018). Difficulties in financial and digital functioning can also be combined since, increasingly, digital skills and using digital tools are required to manage finances (Dury et al., 2018). Acknowledging individuals' financial capacities and digital skills to assess and manage frailty seems important.

In defining the opposite concepts of frailty, autonomy, vitality, and resilience were essential. Older adults described autonomy as the ability to do what they prefer and to choose how they spend their time. Moreover, being vital is very plausible despite (physical) limitations, according to older people. Although vitality originated from physical, social, and psychological aspects (Rozanski, 2023), the psychological aspect of vitality received the most attention from participants. Being motivated and able to fill one's life as one prefers, even though one needs to adjust to declining physical and mental fitness, seemed crucial. This finding aligned with the concept of vitality (Strijk et al., 2015), in which physical and mental energy, motivation to set and reach personal goals, and resilience were seen as the pillars of the concept. In addition, vitality might enhance resilience; having a strong sense of vitality provides the energy to cope with and regulate emotions (Rozanski, 2023). Furthermore, these findings confirmed a positive approach to frailty and ageing in which resilience in older people is emphasized (De Donder et al., 2019).

Our findings regarding the concepts that oppose frailty yielded many different terms and concepts, such as autonomy, resilience, and independence. In frailty literature, there is debate about whether frailty can be placed alongside a continuum and what the endpoints of that continuum are. Experts in the field, for example, recognize a continuum ranging from non-frail to very frail (Gobbens et al., 2010c). In a recent Dutch study, researchers described a continuum from fit or vital to frail (De Breij et al., 2021). However, considering the diverse terms that older people in our study mentioned when asking about the opposite of frailty, we suggest that frailty cannot be placed on a single continuum with concepts such as vitality or resilience. We revealed that older peoples’ perspectives differ from the literature in what is placed opposite to frailty. Therefore, we propose that a frailty continuum can exist alongside related continuums, such as vitality, resilience, or autonomy.

### 7.1 Strengths and limitations

This study shows some strengths. First, the two primary researchers interviewed a large group of older people, resulting in rich and in-depth data on frailty and its opposites. Second, in addition to asking the participants about their perspectives on frailty, we also administered a frailty instrument to determine their frailty status. This informed the analysis and interpretation of the findings. Third, the primary researchers were in close contact during the design of the study, conducting the interviews, and during the analysis. Weekly meetings provided transparency and increased the reliability of the study by discussing the progress and difficulties. Choices were made in collaboration, and the research team was consulted if needed in case of disagreement. This led to considered choices regarding the progress, for example, in recruiting additional participants or analytical choices regarding the themes.

In addition, some limitations have been evaluated. The sample consisted of mainly highly-educated, well-spoken older people, and cultural background was not considered. To enrich our data, we extended the sample by additional recruitment of participants with more diverse backgrounds. However, the sample remained predominantly homogeneous. Therefore, the results should be interpreted with some caution; transfers to wider community settings or other countries will require additional research.

### 7.2 Implications for practice and future research

From the findings, we implied that the current frailty models and the domains they distinguish might be too narrow. Older people perceive that frailty entails more than current models imply, such as the importance of a cognitive dimension, financial and digital aspects, and loss and sacrifice. For future research, it seems important to incorporate these (contextual) factors to create a more comprehensive picture of frailty.

The opposites of frailty enable a focus of care professionals on resilience and vitality in older people. Older people might be frail in a certain domain (e.g., physical) but can be able to cope with their frailty by focusing on their psychological capabilities. Attention to the positive aspects creates opportunities to explore these capabilities of older people so that they can feel acknowledged and valued. Therefore, professionals, such as social workers and nurses, should focus on the (psychological) capabilities of older people. This raises opportunities to manage frailty and its consequences constructively to maintain or restore the well-being of older people.

### 7.3 Conclusion

We explored the concept of frailty and its opposites from the perspective of community-dwelling older people. It appeared that the concept of frailty and its opposites share similar aspects, including physical, psychological, and social dimensions. However, participants shared an even broader view on frailty in which cognition was found to be an essential aspect of frailty. The opposites of frailty were described as autonomy, vitality, and resilience, implicating psychological aspects as a crucial part of the opposites of frailty. These descriptions emphasize the positive aspects of managing frailty in old age. The findings could lead policymakers and professionals to adopt and adhere to older people's views about frailty and its opposing concepts. Connecting policy and care to older people's perceptions might help them to feel involved and confident in taking control of their lives and care trajectories.

## CRediT authorship contribution statement

**Rianne DJ Golbach:** Writing – review & editing, Writing – original draft, Project administration, Methodology, Investigation, Formal analysis, Data curation, Conceptualization. **Nanda Kleinenberg-Talsma:** Writing – review & editing, Writing – original draft, Resources, Project administration, Methodology, Investigation, Formal analysis, Data curation, Conceptualization. **Fons van der Lucht:** Writing – review & editing, Supervision, Methodology, Conceptualization. **Johannes SM Hobbelen:** Writing – review & editing, Supervision, Methodology, Conceptualization. **Harriët Jager-Wittenaar:** Writing – review & editing, Supervision, Methodology, Conceptualization. **Evelyn J Finnema:** Writing – review & editing, Supervision, Methodology, Conceptualization.

## Declaration of competing interest

The authors declare that they have no known competing financial interests or personal relationships that could have appeared to influence the work reported in this paper.
